# The role of syllables in sign language production

**DOI:** 10.3389/fpsyg.2014.01254

**Published:** 2014-11-13

**Authors:** Cristina Baus, Eva Gutiérrez, Manuel Carreiras

**Affiliations:** ^1^Laboratoire de Psychologie Cognitive, Centre National de la Recherche Scientifique (CNRS), Université d'Aix-MarseilleMarseille, France; ^2^Deafness, Cognition and Language Research Centre, University College LondonLondon, UK; ^3^BCBL - Basque Research Center on Cognition, Brain and LanguageDonostia, Spain; ^4^IKERBASQUE, Basque Foundation for ScienceBilbao, Spain; ^5^Departamento de Lengua Vasca y Comunicación, Universidad del País VascoDonostia, Spain

**Keywords:** sign language, speech production, syllables, sign parameters, picture naming

## Abstract

The aim of the present study was to investigate the functional role of syllables in sign language and how the different phonological combinations influence sign production. Moreover, the influence of age of acquisition was evaluated. Deaf signers (native and non-native) of Catalan Signed Language (LSC) were asked in a picture-sign interference task to sign picture names while ignoring distractor-signs with which they shared two phonological parameters (out of three of the main sign parameters: *Location, Movement*, and *Handshape*). The results revealed a different impact of the three phonological combinations. While no effect was observed for the phonological combination Handshape-Location, the combination Handshape-Movement slowed down signing latencies, but only in the non-native group. A facilitatory effect was observed for both groups when pictures and distractors shared Location-Movement. Importantly, linguistic models have considered this phonological combination to be a privileged unit in the composition of signs, as syllables are in spoken languages. Thus, our results support the functional role of syllable units during phonological articulation in sign language production.

## Introduction

In recent years, research on sign language has accumulated evidence to suggest that spoken and sign languages are governed by similar cognitive mechanisms and underpinned by similar neuroanatomical substrates. For instance, the existence of the same linguistic phenomena in both modalities has been taken as evidence that levels of linguistic processing (semantic, lexical, and phonological) are modality-independent. The same semantic, lexical, and phonological effects reported in the spoken modality have been replicated in the sign modality (e.g., Emmorey and Corina, 1990; Corina and Knapp, 2006; Baus et al., 2008; Carreiras et al., 2008; Gutierrez et al., 2012a,b; Hosemann et al., 2013; see Carreiras, 2010 for a review). Furthermore, the same left-lateralized brain network has been described to underlie the processing of signed and spoken languages (e.g., San Jose-Robertson et al., 2004; Emmorey et al., 2007; see also MacSweeney et al., 2008 for a review).

Signs, as well as words, can be decomposed into minimal phonological constituents or formational parameters (Emmorey, 2002; but see Johnston and Schembri, 1999). Three have been considered the main formational parameters of signs (Stokoe, 1960): the *Location* of the sign in relation to the body, the *Movement* of the hand/s and the *Handshape*. Importantly, different studies suggest that these parameters play a different role during language processing (see also current phonological models in sign language; e.g., Brentari, 1998). For instance, using a picture-sign interference task, Baus et al. (2008) reported that lexical access was facilitated when the sign corresponding to the picture and the distractor-sign shared the Handshape, while it was hampered when the Location was shared (see also, Corina and Hildebrandt, 2002; Carreiras et al., 2008; Gutierrez et al., 2012b, for similar results in sign comprehension; see Caselli and Cohen-Goldberg, 2014, for a computational model). Despite the importance of these results, sign production research is still very scarce and hence more evidence is necessary to characterize the role of these phonological parameters and the possible interactions among them. In the present study, we aimed to understand better the processes underlying sign production by asking whether phonological constituents (Location, Movement, and Handshape) are combined into higher order units before a sign is articulated, as phonemes are combined into syllables in spoken languages. To that end, the impact of the different combinations of phonological parameters on sign production was explored.

In spoken languages, syllables are considered the functional units during speech planning (e.g., Levelt and Wheeldon, 1994; Carreiras and Perea, 2004; Cholin et al., 2006; Laganaro and Alario, 2006). Accordingly, models of speech production describe the locus of syllables within the production system, either at the word-form encoding level (phonological syllables, see Dell, 1988) or during articulatory preparation (e.g., Crompton, 1981; Levelt and Wheeldon, 1994). Experimental evidence for the existence of syllables in speech production comes from different sources, such as speech errors or syllabic effects. For instance, it has been shown that speech errors respect the *syllable position constraint* (e.g., Boomer and Laver, 1968; Mackay, 1970). That is, for those sound/form exchanges occurring between close-by words (such as rack pat for pack rat), onsets are exchanged with onsets but not with codas. Moreover, the role of syllabic units in word production has been explored mainly through two effects: the so-called *syllabic frequency effect* and the *syllabic priming effect*. The syllabic frequency effect refers to the observation that speakers are faster at naming words (and pseudowords) containing high frequency syllables than low frequency ones (e.g., Levelt and Wheeldon, 1994; Aichert and Ziegler, 2004; Alario et al., 2004; Carreiras and Perea, 2004; Cholin et al., 2006; Laganaro and Alario, 2006). The syllabic-priming effect refers to the observation that speakers are faster at naming a word (e.g., basis) when it has been primed with a syllable (ba) that respects the syllable boundaries of the word, than with an incongruent syllable (bas) that does not respect such boundaries (e.g., Ferrand et al., 1996, 1997; but see, Baumann, 1995; Schiller, 1998; Schiller et al., 2002; Schiller and Costa, 2006, for failed attempts to replicate the syllabic priming effect).

Linguistic theories of the structure of signed language agree on the existence of such syllabic-like units in signed language. That is, the syllable as a formal concept has an analog in signed language. The parallelism between syllables in spoken and signed languages stems from the idea that the way phonological constituents are organized into syllables depends on the sonority of the segments (Perlmutter, 1992). Signs are sequentially organized in terms of static-dynamic alternation that could be compared to consonants (holds) and vowels (movements) in the spoken modality. Syllables must include a *nucleus*, which corresponds to the maximal peak of sonority, the vowel, and may include an *onset* or a *coda* (Selkirk, 1982). The same applies to sign language. Models of sign language tend to attribute to the Movement the status of the nucleus (e.g., Chinchor, 1978; Brentari, 1990; Corina, 1990; Sandler, 1993; Brentari, 1998). In fact, Sandler's Location-Movement-Location model (Sandler, 1987, 1989) proposes that it is the combination of Locations and Movements that composes a syllable (see Chinchor, 1978; Wilbur, 1993, for a fairly different view). Indeed, the Movement is considered the visual equivalent of “*sonority*,” being then the most salient parameter, which can be easily differentiated from the other parameters. For instance, as do vowels in the spoken modality, Movements in a sign carry prosodic as well as emotional information. Moreover, for some signs, the number of Movement repetitions determines whether a given sign is a noun or a verb (e.g., GLASS and TO DRINK in LSC have the same C Handshape next to the mouth with one repetition of the sign glass and two for to drink). Importantly however, as indicated by Emmorey et al. (2007), the fact that words and signs can be decomposed into similar syllabic-units is not a guarantee that syllabification processes are the same in word and sign production. There are several differences between spoken and signed languages that could contribute to the suggested difference in processing (for instance, most signs are monosyllabic, Brentari, 1990). Indeed, the same happens if we consider the role of syllables across different spoken languages. For instance, while syllables exist across languages, their impact as segmentation units is stronger for those languages with clear syllabic boundaries (e.g., Romance languages). Moreover, planning units might vary depending on the task in hand. In Chinese, while syllables are the functional unit during speech production (Chen et al., 2002), logographemes are the proximal unit in handwritten production (Chen and Cherng, 2013). Thus, even if syllables have been linguistically described in signed language, it is important to describe their psychological reality by exploring how signers process these syllabic units in sign language (see Corina et al., 2014).

To date however, the functional role of syllables in sign language processing has scarcely been investigated (Corina and Knapp, 2006; Dye and Shih, 2006; Mayberry and Witcher, 2006; Gutierrez, 2008). Interestingly, these few results point to a special status of the combination of Location-Movement in both sign comprehension and production. For instance, Dye and Shih (2006) tested the speed with which deaf signers took lexical decisions in a priming paradigm in which primes and targets shared two out of the three phonological parameters (Location, Handshape, and Movement). Their results revealed that native deaf signers were faster at making decisions on the target, exclusively when prime and target shared Location and Movement. Similarly, Corina and Knapp (2006) reported a facilitatory effect for the combination Location-Movement in ASL sign production using a picture-sign interference task. However, although these results provide evidence of the privileged status of this phonological combination in sign production, they remain silent about the role of the other phonological combinations. Thus, the present study aimed to further investigate how the different combinations of parameters, namely Location-Movement, Location-Handshape and Handshape-Movement affect the speed with which signs are produced.

Our second aim was to expand Corina and Knapp's results (2006) by exploring the influence of age of acquisition on the processing of these syllabic-like units. Age of acquisition is a very interesting issue to address here, since signed language offers the unique opportunity to test age of acquisition differences in first language processing. Several studies have reported differences in performance between signers who acquire a sign language early relative to those who acquire sign language later in life (Mayberry and Fisher, 1989; Newport, 1990; Corina and Hildebrandt, 2002; Carreiras et al., 2008; Gutierrez et al., 2012a). Such differences have been attributed to a “phonological bottleneck” by which the form-based properties of signs are processed less efficiently the later the sign language is acquired. For instance, in Dye and Shih (2006), no phonological effect was observed for the Location-Movement combination when non-native signers were tested in the priming experiment. Instead, priming effects arose uniquely when primes and targets shared the Movement parameter in isolation. To further explore how age of acquisition influences lexical access during sign production, we compared the performance of two groups of signers that differed in the age at which sign language was acquired. The hypothesis is that if non-native signers are less efficient in processing phonological units in sign language, it is possible that the different phonological combinations do not equally impact native and non-native processing.

In the present study we used a picture-sign interference task (Corina and Knapp, 2006; Baus et al., 2008) and asked deaf signers who had acquired the signed language early (born within deaf families) or late (after the age of 10) to sign the corresponding picture-sign while ignoring a distractor. The task was an adaptation of the picture-word interference paradigm, which has been extensively used in the language production literature to reveal the functional dynamics of lexical retrieval processes in speech production. Note that this is not to say that comprehension mechanisms are not involved in the processing of the distractors.

## Methods

### Participants

Twenty-four deaf signers participated in this study (11 women). The participants were the same as in Baus et al. (2008). All of them were deaf from birth and used Catalan Sign Language (LSC) on a daily basis as preferred means of communication. Twelve participants were considered as native signers (age range 18–51, mean age 30.3, *SD* = 7.6). They were born in deaf families (parents or older siblings) and acquired the signed language before the age of 5. The remaining were non-native signers (from hearing families) (age range 18–44, mean age 26.4, *SD* = 5.8) who learned LSC at the mean age of 12 (age of exposure range 10–31 years, mean = 16, *SD* = 7.2). Both groups of participants had attended “oralist” schools (it is relatively new to find schools adapted to the deaf community). All of them had completed the years of compulsory education (primary school, up to 14 years old), with only a few of them completing the secondary levels of education (5 participants). All participants reported feeling more comfortable using the signed than the spoken language.

### Materials

Thirty line-drawings depicting simple objects from different semantic categories were selected (e.g., Snodgrass and Vanderwart, 1980). For each picture, two video-signs (distractors) were created: one phonologically related and one unrelated. In the phonologically related condition, the sign corresponding to the picture and the distractor-sign shared two out of the three main parameters. Thus, there were three types of phonological overlap: Handshape-Movement, Location-Handshape, and Location-Movement (ten items per condition). Given that the pool of picturable stimuli is limited, it was not possible to pair each picture-sign with a distractor-sign of each phonological condition. Thus, each picture was assigned to just one of the phonological conditions and was paired with one phonologically related and one phonologically unrelated distractor. In the unrelated condition, the picture's corresponding sign and the video-sign had no phonological or semantic relationship.

**Figure 1:**
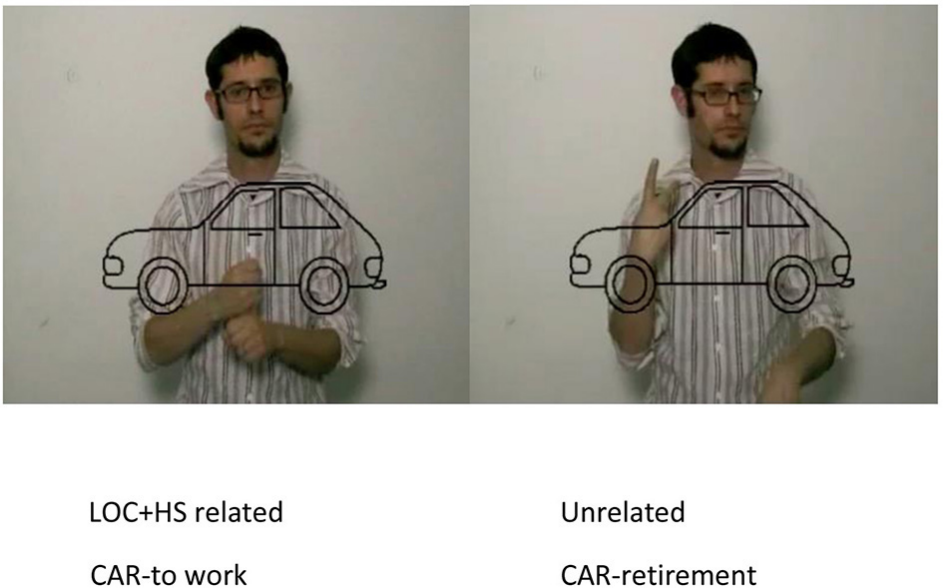
**An example of the stimuli employed in the experiment**. The sign corresponding to the picture CAR is formed by the A- Handshape, located in the neutral space and with a movement that resembles the action of moving the steering wheel. This sign shares the Location (LOC) and the Handshape (HS) with the sign TO WORK (left image) and does not share any of these parameters with the sign RETIREMENT (right image).

During the experiment, participants saw each picture twice, once in a phonologically related pair and once in an unrelated pair. The order of appearance was randomized. The results were then based on the comparison between the related and the unrelated conditions, where the same picture was used (see Figure 1 for an example and the Appendix for the full list of materials in the Supplementary Material) and not on the comparison between the different phonological combinations. The pictures appeared superimposed on a video of a deaf person signing and were presented to participants at the same time (SOA 0).

All videos had an approximate duration of 500 ms and comprised both the video distractor and the picture, that is, the picture appeared simultaneously with the onset of the distractor video sequence and remained visible on the screen together with the last frame of the video distractor until participants responded.

### Procedure

Participants were tested in a quiet room, avoiding visual distractors. Before the experiment started, instructions were signed to the participant in LSC. They were instructed to sign the name of the picture while ignoring the video presented at the back. After ensuring that participants understood the instructions, they were presented with a booklet containing all the pictures of the experiment to ensure that they used the designated sign during the experiment. Participants were then familiarized with the task in 10 practice trials with similar characteristics to the experimental ones.

During the experiment, the structure of the trial was as follows: (1) an instruction indicating that a new trial was about to start appeared on the screen, indicating that participants should press the two response buttons (in the response box) with their two hands and hold them pressed until their response; (2) while they pressed the response buttons, an asterisk appeared in the center of the screen for 500 ms, followed by a blank interval of 300 ms; (3) a video appeared containing the video-distractor and the picture (see Figure 1) and lasted for approximately 500 ms. When the video finished, the image remained still on the last frame until the participant's response; (4) 2000 ms after the participant's response, the message telling the participant to press the button responses appeared again. Reaction times were registered from the onset of the picture + video presentation to the moment the participant raised her hands off the button box to sign the name of the picture. Stimulus presentation and reaction times were controlled by Psyscope software (Cohen et al., 1993). Participants were videotaped during the experimental session to score for errors.

## Results

Responses different from the ones designated by the experimenter were considered as production errors and were excluded from the latency analyses. Moreover, those responses in which the participant stopped before signing were considered as hesitations and therefore counted as errors. Two pictures were also excluded because more than 80% of the participants used a sign different from the one designated by the experimenter (one picture in the Location-Handshape and one in the Location-Movement condition; indicated by an asterisk in the Appendix—Supplementary Material). Finally, signing latencies above or below two standard deviations in each condition were also excluded. Data trimming led to final exclusion of 10% of the data from the latency analysis.

Median latencies and error rates were analyzed for each phonological condition separately (Handshape-Movement, Location-Handshape, and Location-Movement). Note that using the median instead of the mean is a common practice in the analysis of populations in which a lot of variability and extreme values can be encountered.

In a 2 × 2 ANOVA, the phonological relationship (related vs. unrelated) and the group of participants (native vs. non-native) were entered as within participant and between items factors, respectively. The analyses considering the error rates did not reveal any significant results (all *p*'s > 0.2) and they are not further discussed. Moreover, native and non-native signers did not differ in their overall signing performance. The main effect of group was not significant in any of the conditions explored (all *F*'s < 1).

Regarding signing latencies (Table 1), participants were slower signing those pictures sharing Handshape and Movement with the video-distractor than the same pictures when the distractor was phonologically unrelated [*F*_1(1, 22)_ = 7.47, *p* < 0.05 and *F*_2(1, 18)_ = 4.34, *p* = 0.05]. That is, the Handshape-Movement phonological combination revealed an interference effect. Moreover, the interaction between phonological relatedness and group of participants (Natives vs. Non-natives) was significant in the analysis by participants [*F*_1(1, 22)_ = 4.04, *p* = 0.05 and *F*_2(1, 18)_ = 1.29, *p* = 0.27]. *Post-hoc* comparisons indicated that the non-native group was affected by the Handshape-Movement phonological overlap between the picture and the distractor [*F*_1(1, 22)_ = 11.2, *p* < 0.01], but not the native group (*F* < 1).

**Table 1:** **Median reaction times (RT) and percentage of errors (%error) in each phonological condition for the native and non-native group of participants**.

**Type of relationship**		**Natives**			**Non-natives**		
		***RT***	***SD***	**% Error**	***RT***	***SD***	**% Error**
HM	Related	606	224	3.8	535	169	8.3
	Unrelated	596	230	4.5	474	142	5.8
	HM effect	10			59		
LH	Related	597	263	2.8	508	157	2
	Unrelated	577	215	2.5	491	152	2.7
	LH effect	20			17		
LM	Related	565	237	4.6	449	124	6.1
	Unrelated	592	256	6.5	509	180	3.0
	LM effect	−27			−50		

*HM, Handshape-Movement; LH, Location-Handshape; LM, Location-Movement*.

For the Location-Handshape condition (LH), there were no significant differences between the signing latencies in the related and the unrelated conditions [*F*_1(1, 22)_ = 1.69, *p* = 0.20 and *F*_2(1, 16)_ = 1.72, *p* = 0.20]. Moreover, as indicated by the lack of interaction with age of acquisition (*F* < 1), neither native nor non-natives were affected by the Location-Handshape phonological overlap.

Finally, we found a main effect of the Location-Movement combination [*F*_1(1, 22)_ = 5.61, *p* < 0.05 and *F*_2(1, 16)_ = 4.41, *p* < 0.05]. Participants were faster signing pictures when sharing the Location and the Movement with the distractor than when signing the same pictures when presented with an unrelated distractor. Both groups of participants benefited from the Location-Movement phonological overlap between target and distractor, as indicated by the lack of interaction between the phonological condition and group of participants (*F* < 1).

## Discussion

This study aimed to explore the role of the different syllabic units during sign production. Specifically, we tested whether the combination of Location and Movement, suggested by sign language models as the most important syllabic unit, would stand out during on-line LSC sign production in comparison to other parameter combinations.

Our results were clear-cut: both native and non-native signers were faster at signing the intended target only when it was presented together with a distractor that shared the Location and the Movement^1^. In line with previous research (Corina and Knapp, 2006), the present results support the idea that the combination of parameters Location-Movement seems to enjoy a privileged status during sign production, as well as during sign comprehension (e.g., Dye and Shih, 2006). Indeed, linguistic models of sign structure have described Movements and Locations as the main syllabic building blocks (e.g., Sandler, 1987; Corina and Emmorey, 1993; Brentari, 1998) with Handshapes being represented on a separate structural tier (e.g., Sandler, 1993). Although those models were created to describe signs in American Signed Language (ASL), our results and others suggest a more general effect of the Location-Movement combination across the world's signed languages, at least in what concerns Spanish Signed language (Gutierrez, 2008), British Signed Language (Dye and Shih, 2006), and Catalan Signed language. Note, however, that with these results we cannot attribute to the Location-Movement combination the unique status of syllabic unit in signed language. The reason is that finding that the Location-Movement combination influences sign production does not demonstrate that other syllabic structures do not exist in sign language (e.g., Chinchor, 1978). For instance, the Handshape-Movement combination also influenced sign production (although in the opposite direction) of non-natives, suggesting a different impact of the three phonological combinations rather than the unique existence of Location-Movement as syllabic unit. Thus, the interesting question for us is: What is special about the Location-Movement combination in sign language processing? If we consider that the inventory of Locations and Movements within signed languages is significantly smaller than the inventory of Handshapes, one possibility is that particular Locations and Movements appear more frequently in the lexicon than Handshapes do. Indeed, children acquire control of the Location and Movement parameters much earlier than they master Handshapes, which require specialized dexterity of the hands and fingers (e.g., Siedlecki and Bonvillian, 1993; Conlin et al., 2000; Marentette and Mayberry, 2000). Furthermore, there is evidence that when signers make an error, the probability of involving a change in Movement or Location is relatively low (8%) compared to the probability of making an error that involves a change in the Handshape (82%; Hohenberger et al., 2002; see also Orfanidou et al., 2009). Similarly, Location and Movement are less prone to errors than Handshape in aphasic signers (Corina et al., 1992; Corina, 2000). Thus, it could be argued that our results are due to Location and Movement being more strongly represented than Handshape. However, this idea is not longer tenable if we compare the influence of these parameters when presented in isolation or jointly. Many studies have reported a facilitatory effect when Location and Movement are presented jointly, both in sign comprehension and production, and regardless of the age at which sign language was acquired. In contrast, the effect of each parameter when presented in isolation is highly variable. For instance, both inhibitory (Baus et al., 2008; Carreiras et al., 2008; see also, Caselli and Cohen-Goldberg, 2014; for a computational model on the location effects) and facilitatory effects (e.g., Dye and Shih, 2006; Orfanidou et al., 2009) have been reported when in the same task Location was manipulated in isolation. Thus, our results suggest that phonological combinations involving Location-Movement are indeed an important functional unit in lexical access and not just the additive effect of sharing two parameters (Wilbur and Allen, 1991).

Phonological combinations involving Location and Movement in sign languages have been considered to be more perceptually salient than those involving Handshape (e.g., Klima and Bellugi, 1979; Corina and Emmorey, 1993; Hohenberger et al., 2002). For instance, Hildebrant and Corina (2002) asked participants to judge the phonological similarity between a target-sign and surrounding flanker-signs, which could share the Handshape-Movement, the Location-Handshape or the Movement-Location parameters. Native signers rated those flankers that shared the Location-Movement combination more similar to the target than those involving the Handshape. Our results are in line with the idea of Location-Movement being the most salient sub-lexical (syllabic or not) unit in sign production. In this context, accessing the phonological codes composing the picture's corresponding sign would be faster for those signs sharing Location and Movement, since they will be judged as more similar than the other two phonological combinations. This would support the idea that linguistic distinctions are based on salient perceptual distinctions (Corina et al., 2014). Alternatively (but not mutually exclusive), our results could be interpreted as an effect of the *frequency* with which the parameters co-occur in sign language, with sign-units involving Location and Movement appearing more frequently than those involving Handshape. Our results would be in line with those studies in the spoken modality showing that speakers are faster at naming words containing high-frequency syllables (which they have produced more often) than words containing low-frequency ones (e.g., Carreiras and Perea, 2004; Cholin et al., 2006; Laganaro and Alario, 2006). However, here we cannot exclude the possibility that other sublexical variables, such as the *biphone frequency* (frequency with which two phonemes co-occur regardless of whether they respect the syllabic boundaries or not), are responsible for the observed effect. In the spoken modality, the speed with which a word is produced is influenced both by the syllabic and the biphone frequency (Vitevitch et al., 2004). Such distinction has not been described in the signed modality, possibly due to the simultaneous perception of parameters within a sign. Thus, whether Location-Movement is the most frequent syllabic unit or just comprises the sequences that co-occur with more probability in the language cannot be determined from the present results. Lee and Goldrick (2008) also argued that speakers are not only sensitive to the frequency with which sub-syllabic sequences occur within a language but also to the strength of association. Importantly, if the language of the speaker determines the preference for one sequence (for instance, in Korean, sequences involving onset-vowels are strongly associated, whereas in English it is vowel-coda sequences which are more associated), it is possible that our results reveal the preference of signers for those sequences strongly associated in sign language, namely Location-Movement sequences. At present, we cannot determine whether the origin of the observed effect stems from Location-Movement being the most salient structure or the phonological sequence more probable in the language, but this opens interesting questions for future studies on phonological processing in signed language.

Finally, regarding the question of how the age of sign language acquisition might influence its phonological processing, we did not find differences between groups for the Location and Movement combination. However, the two groups differed in two aspects. Firstly, there was a tendency for shorter latencies in the non-native group than in the native one. This result was unexpected if we consider previous evidence pointing to less efficient phonological processing in non-native signers (e.g., Gutierrez et al., 2012a). Nevertheless, the fact that such differences were not significant, together with the observation that the non-native signers were overall younger than the native signers and that this is known to have an impact on processing-speed (e.g., Salthouse, 1993), prevent us from making further interpretations. Secondly and more interesting, we only obtained a difference between the two groups for the Handshape-Movement combination. Non-native signers were slower at signing pictures in the presence of the Handshape-Movement phonological distractor than in the presence of an unrelated distractor. This piece of evidence supports the idea that the late acquisition of signs results in subtle differences in sign language processing (Newport, 1990; Mayberry and Eichen, 1991; Neville et al., 1997; Corina and Hildebrandt, 2002; Newman et al., 2002; Carreiras et al., 2008; Morford et al., 2008) often involving a qualitatively different processing of Handshapes (Emmorey et al., 2003; Carreiras et al., 2008; Orfanidou et al., 2009; Best et al., 2010; Gutierrez et al., 2012a). For instance, Hildebrant and Corina (2002) found that non-native signers judged signs as perceptually more similar when sharing the Handshape than the other parameters, while native signers based their decision on the Movement. However, Handshape cannot be the only explanation for two reasons: (1) a facilitatory effect of Handshape was reported by Baus et al. (2008) while the manipulation of Handshape in combination with Movement led to an interference effect, and (2) if Handshape is the parameter responsible for the pattern of results found for non-natives, similar results would be expected for the other phonological combination involving Handshape, namely the Location-Handshape condition. Considering these and previous findings, the pattern of results reported for non-natives is rather complex, even when more sensitive techniques such as ERPs have been employed (Gutierrez et al., 2012a). For instance, Gutierrez et al. (2012a) found that non-natives were not affected by Handshape relatedness during sign recognition either of signs or non-signs, while previous studies have reported Handshape to be the most salient phonological parameter for late signers (Corina and Hildebrandt, 2002). Thus, at this point, any interpretation of the interference effect observed for Handshape-Movement would be very tentative and premature, but it opens an excellent question to pursue in the future. Importantly, this effect also supports the idea indicated above that two-parameter effects are not just the additive effect of the two single parameters.

## Conclusion

In sum, our results provide clear evidence of the special role that certain phonological combinations play in sign language production. Location-Movement is the only phonological combination that enjoys a benefit in processing during sign production.

### Conflict of interest statement

The authors declare that the research was conducted in the absence of any commercial or financial relationships that could be construed as a potential conflict of interest.

## Footnotes

^1^Note that although signing latencies are measured in the picture-signing interference task, both comprehension and production mechanisms are involved when performing such task.
