# Supplementary material for: The role of syllables in sign language production
Source: Front Psychol. 2014 Nov 13;5:1254. doi: 10.3389/fpsyg.2014.01254 (PMC4230165; doi:10.3389/fpsyg.2014.01254)
Supplement: Supplementary file 1 [file DataSheet1.DOCX]

|  | **HS+MOV** |  |
| --- | --- | --- |
| *picture* | **RELATED** | **UNRELATED** |
| TRAY | tiger | proud |
| BUS | communication | twenty |
| SHIRT | bikini | old |
| FACE | only | Monday |
| CHERRY | two | Tuesday |
| SHEEP | crazy | choice |
| SKIRT | ruff | Santander*(Spanish city)* |
| COCK | elegant | official |
| NOSE | Thursday | bird |
| WATCH | also | caravan |
|  | **LOC+HS** |  |
| picture | **RELATED** | **UNRELATED** |
| HOUSE | divorce | Girona*(Spanish city)* |
| CAR***** | to work | retirement |
| HELICOPTER | mushroom | Saturday |
| RAIN | kitchen | whistle |
| POTATO | hang | parrot |
| SAW | brother-in-law | foreign |
| CHAIR | frog | goat |
| TELEVISION | painting | lamb |
| SCISSORS | fish | to build |
| TELEPHONE | blue | seal |
|  | **LOC+MOV** |  |
| picture | **RELATED** | **UNRELATED** |
| TREE | lawyer | Roman |
| BOX | stamp | February |
| KANGAROO | ship | wrong |
| CROSS | to share | eye |
| BREAD | month | Sunday |
| COMB | fireman | same |
| FORK***** | party | Egypt |
| BULL | police | wedding |
| WINDOW | dirty | meat |
| SHOE | to explain | yellow |

APPENDIX. List of stimuli employed in the Experiment (the asterisks indicate the stimuli excluded from the analysis).
